# Borderline personality disorder and childhood trauma: exploring the affected biological systems and mechanisms

**DOI:** 10.1186/s12888-017-1383-2

**Published:** 2017-06-15

**Authors:** Nadia Cattane, Roberta Rossi, Mariangela Lanfredi, Annamaria Cattaneo

**Affiliations:** 1 Biological Psychiatry Unit, IRCCS Istituto Centro San Giovanni di Dio - Fatebenefratelli, via Pilastroni 4, Brescia, Italy; 2 Psychiatry Unit, IRCCS Istituto Centro San Giovanni di Dio - Fatebenefratelli, via Pilastroni 4, Brescia, Italy; 3 Stress, Psychiatry and Immunology Laboratory, Department of Psychological Medicine, Institute of Psychiatry, King’s College London, 125 Coldharbour Lane, London, SE5 9NU UK; 4 Department of Psychological Medicine, Institute of Psychiatry, Psychology and Neuroscience, King’s College London, 125 Coldharbour Lane, London, SE5 9NU UK

**Keywords:** Borderline personality disorder, Childhood trauma, HPA axis, Endogenous opioid system, Neurotransmission, Neuroplasticity, Neuroimaging studies, Epigenetic mechanisms

## Abstract

**Background:**

According to several studies, the onset of the Borderline Personality Disorder (BPD) depends on the combination between genetic and environmental factors (GxE), in particular between biological vulnerabilities and the exposure to traumatic experiences during childhood. We have searched for studies reporting possible alterations in several biological processes and brain morphological features in relation to childhood trauma experiences and to BPD. We have also looked for epigenetic mechanisms as they could be mediators of the effects of childhood trauma in BPD vulnerability.

**Discussion:**

We prove the role of alterations in Hypothalamic-Pituitary-Adrenal (HPA) axis, in neurotrasmission, in the endogenous opioid system and in neuroplasticity in the childhood trauma-associated vulnerability to develop BPD; we also confirm the presence of morphological changes in several BPD brain areas and in particular in those involved in stress response.

**Summary:**

Not so many studies are available on epigenetic changes in BPD patients, although these mechanisms are widely investigated in relation to stress-related disorders. A better comprehension of the biological and epigenetic mechanisms, affected by childhood trauma and altered in BPD patients, could allow to identify “at high risk” subjects and to prevent or minimize the development of the disease later in life.

## Background

Borderline Personality Disorder (BPD) is a pervasive pattern of emotional dysregulation, impulsiveness, unstable sense of identity and difficult interpersonal relationships [1]. The prevalence rates of BPD are between 0.2–1.8% in the general community, 15–25% among psychiatric inpatients and 10% of all psychiatric outpatients [2, 3]. Among the different aetiopathological theories that have been proposed over years, the most supported is the one proposed by Linehan in 1993 [4], which suggests that BPD can be the result of the interactions between biological and psychosocial factors [2], in particular between biologically based temperamental vulnerabilities and adverse and traumatic experiences during childhood.

BPD is a disorder primarily characterized by emotion dysregulation and indeed, patients with BPD show heightened emotional sensitivity, inability to regulate intense emotional responses, and a slow return to emotional baseline. Linehan proposed also that the development of BPD occurs within an invalidating developmental context characterized by intolerance toward the expression of private emotional experiences during childhood [4]. As a consequence, children exposed to this adverse environment show inability to learn how to understand, label, regulate, or tolerate emotional responses and, conversely, they vacillate between emotional inhibition and extreme emotional lability.

Recently, Hughes and colleagues [5] have proposed an integration to the aethiopathogenetic model of BPD, emphasizing the role played by a lack of social proximity or responsiveness from relevant caregivers in the development of BPD symptoms, which in turn impairs the individual’s emotion regulation. Affect regulation difficulties have been also proposed as key mediators in the relationship between childhood trauma and BPD [6].

Several studies have shown that a diagnosis of BPD is associated with child abuse and neglect more than any other personality disorders [7, 8], with a range between 30 and 90% in BPD patients [7, 9].

Adverse childhood experiences are also related to BPD symptom severity [9–11]. In support to this, Widom and collaborators [12] followed 500 children who had suffered physical and sexual abuse and neglect and 396 matched controls, and they observed that significantly more abused/neglected children met criteria for BPD in adulthood in comparison to controls. However, the presence of a risk factor, such as adverse childhood events, was not necessary or sufficient to explain the reason why some individuals developed BPD symptoms in adulthood, whereas others did not.

In a recent study, Martin-Blanco and collaborators [10] have hypothesized that the interaction of childhood trauma and temperamental traits could be associated with the severity of BPD. In this regard, they have evaluated the self-reported history of trauma, the psychobiological temperamental traits and the severity of the BPD symptoms in a cohort of 130 BPD patients. Data showed a correlation only between childhood maltreatment and sociability and no other correlation was observed. Moreover, the interaction between high neuroticism-anxiety traits and the presence of severe emotional abuse was associated with the severity of the disorder.

Symptom overlap has been reported between BPD diagnosis and other disorders including Post-Traumatic Stress Disorder (PTSD) and other axis I disorders [13]. Moreover, in recent decades, different nosographic descriptions have been suggested to characterize the different symptoms associated with trauma, like complex Post-Traumatic Stress Disorder (cPTSD) [14], also known as Disorders of Extreme Stress Not Otherwise Specified (DESNOS) [15], which describes a clinical syndrome following an experience of interpersonal traumatic victimization and shares many similarities with BPD, including pathological dissociation, somatizations, dysregulation of emotions, altered central self and relational schemas. The definition of cPTSD therefore refers to the experience of severe and/or prolonged traumatic situations, and does not merely identify the effects of devastating traumatic events (like violence or chronic maltreatment), which fall under the category of PTSD or Acute Stress Disorder. Indeed, exposure to particular types of traumatic experiences may result in far more insidious and crippling psychopathogenic disorders than PTSD, compromising the sound development of attachment behavior related systems and of the ability to modulate emotions [16]. Recent research is currently trying to determine whether cPTSD and BPD diagnosis in comorbidity with PTSD are distinct or should both be considered and named as trauma-related disorders [17]. A recent review [18] has explored the mechanisms through which childhood trauma is related to the development of BPD in adulthood, and has discussed how interrelated factors (such as heritable personality traits, affect regulation and dissociation, trauma symptoms) could be mediators in the relationship between childhood trauma and BPD.

Based on all these findings, in the following paragraphs we will discuss alterations in several neurobiological systems and in brain morphology that can be induced by exposure to early life adverse experiences and that are also associated with BPD (see Table 1). We will examine the impact of early stressful events on different biological systems and mechanisms, possibly identifying biomarkers that could be involved in BPD vulnerability. This might allow to identify at high risk BPD subjects earlier, and to develop intervention strategies and programs.

**Table 1:** Summary of the papers cited in the review and showing alterations in different biological systems in BPD

Biological systems	Authors	Sample size	Date of study	Main Results
HPA axis	Southwick et al. [26]	37 subjects with PTSD comorbid with BPD; 18 subjects only with PTSD	2003	Higher 24 h urinary cortisol levels in patients with PTSD compared to patients with PTSD and comorbid BPD.
	Wingenfeld et al. [27]	21 female patients with BPD; 24 healthy female controls.	2007	Higher overnight urinary cortisol levels in BPD patients compared to controls. Very high cortisol levels were found only in BPD patients with a low number of PTSD symptoms.
	Rinne et al. [28]	39 BPD patients (24 with and 15 without sustained childhood abuse and comorbid PTSD (*n* = 12) or MDD (*n* = 11)); 11 control subjects	2002	Higher ACTH and cortisol levels in the blood of BPD females who had experienced childhood abuse during the DEX/CRH test.
	Carvalho Fernando et al. [29]	32 female BPD patients; 32 healthy female	2013	Acute cortisol levels decreased the reaction time to target stimuli in both BPD patients and controls.
	Martin-Blanco et al. [30]	481 subjects with BPD; 442 controls	2016	Case-control study focusing on 47 SNPs in 10 HPA axis genes. An association between polymorphic variants within the FKPB5 and the CRHR genes with the diagnosis of BPD was shown. Two FKBP5 SNPs were more frequently represented in patients with a history of childhood trauma.
Neurotransmission	Wagner et al. [42]	159 BPD patients	2009	Association between stressful events and low impulsivity in BPD patients. 5-HTTLPR S-allele carriers showed higher impulsivity scores when exposed to stressful events than LL omozygotes.
	Wagner et al. [47]	112 female BPD patients	2010	COMT Val158Met SNP was associated with early life stressful events and impulsive aggression in female BPD patients
	Wagner et al. [48]	159 BPD patients	2010	The effect of COMT Val158Met SNP on the association between stressful life events and impulsivity was not confirmed.
	Tadic et al. [49]	161 Caucasian BPD patients; 156 healthy controls.	2009	The COMT Met158Met SNP was over-represented in BPD patients compared to controls. No differences in 5-HTTLPR genotype were found. An interaction between the COMT Met158 and the 5-HTTLPR s-allele was observed.
	Martin-Blanco et al. [50]	481 BPD subjects; 442 controls	2015	Genetic variants within COMT, DBH and SLC6A2 genes were associated with an enhanced risk to develop BPD
Endogenous Opioid System	Kalin et al. [57]	8 infant rhesus monkeys (4 males and 4 females)	1988	The endogenous opioid system mediates separate-induced vocalizations and influences the HPA axis activation in rhesus monkeys using the mother-infant separation paradigm.
	Prossin et al. [61]	18 un-medicated female BPD patients; 14 female controls	2010	BPD patients had greater regional μ-opioid availability at baseline in the left necleus accumbens, the hypothalamus and the right hippocampus/parahippocampus relative to controls, showing an endogenous opioid system activation.
Neuroimaging studies	Driessen et al. [36]	21 female BPD patients; 21 female controls	2000	Volume reduction in the hippocampus and in the amygdala in BPD patients compared to controls.
	Schmahl et al. [38]	25 unmedicated female patients with BPD (10 with and 15 without comorbid PTSD); 25 female controls	2009	Hippocampal volume reduction in patients with BPD and comorbid PTSD as compared to controls.
	Kreisel et al. [70]	39 BPD patients; 39 controls	2014	Smaller hippocampal volume in BPD patients with a lifetime history than those without comorbid PTSD.
	Boen et al. [71]	18 women with BPD; 21 controls	2014	Two hippocampal structures (DG-CA4 and CA2–3 subfields) were significantly smaller in patients with BPD than controls.
	Kuhlmann et al. [73]	30 BPD patients; 33 controls	2013	Patients with BPD showed lower hippocampal volumes than controls, but higher volumes in the hypothalamus.
	Rodrigues et al. [63]	124 BPD patients; 147 controls	2011	Both the left and the right sides of the hippocampus were reduced in BPD patients with PTSD when compared to controls.
	Ruocco et al. [37]	205 BPD patients; 222 controls	2012	Bilateral volume reductions of the amygdala and hippocampus were not related to comorbid MDD, PTSD or substance use disorders.
Epigenetics	Martin-Blanco et al. [88]	281 subjects with BPD	2014	An association between NR3C1 methylation levels and childhood trauma was found in blood samples of BPD patients.
	Dammann et al. [89]	26 BPD patients; 11 controls	2011	An increase in the methylation levels of HTR2A,NR3C1,MAOA,MAOB and COMT was found in BPD patients as compared to controls.
	Perroud et al. [91]	346 BD, BPD, and ADHD patients	2016	Differential 5-HT3AR methylation levels were associated with the severity of childhood trauma, mainly found in BPD patients.
	Teschler et al. [93]	24 female BPD patients; 11 female controls	2013	Genome-wide methylation analyses revealed increased methylation levels of several genes (APBA2,APBA3,GATA4,KCNQ1,MCF2,NINJ2,TAAR5) in blood of BPD female patients and controls.
	Prados et al. [94]	96 BPD subjects suffering from a high level of child adversity; 93 subjects suffering from MDD and reporting a low rate of child maltreatment	2015	Several CpGs within or near genes involved in inflammation and in neuronal excitability were differentially methylated in BPD patients compared to MDD patients or in relation to the severity of childhood trauma.
	Teschler et al. [95]	24 female BPD patients; 11 female controls	2016	A significant aberrant methylation of rDNA and PRIMA1 was revealed for BPD patients using pyrosequencing. For the promoter of PRIMA1, the average methylation of six CpG sites was higher in BPD patients compared to controls. In contrast, the methylation levels of the rDNA promoter region and the 5′ETS were significantly lower in patients with BPD compared to controls.
Neuroplasticity	Koenigsberg et al. [109]	24 medication-free BPD patients; 18 healthy control subjects	2012	Decrease of PKC and BDNF protein levels in the blood of BPD patients.
	Tadic et al. [49]	161 Caucasian BPD patients; 156 healthy controls.	2009	Association between HTR1B A-161 variant and the functional BDNF 196A allele in BPD patients.
	Perroud et al. [90]	115 subjects with BPD; 52 controls	2013	Higher methylation levels in BDNF CpG exons I and IV in BPD patients than in controls. Higher BDNF protein levels in plasma of BPD patients than in controls.
	Thaler et al. [92]	64 women with bulimia nervosa and comorbid BPD; 32 controls	2014	Hypermethylation within BDNF promoter region sites in women with bulimia nervosa and with a history of BPD and/or trauma events.

## Discussion

### Neurobiological mechanisms involved in BPD

#### BPD and the hypothalamic-pituitary-adrenal axis

The Hypothalamic-Pituitary-Adrenal (HPA) axis is one of the neuroendocrine systems which mediate the response of the body to stress. Although the stress response mechanism is meant to maintain stability or homeostasis, its long-term activation, as consequence of chronic stress exposure, may have deleterious effects on the body, increasing the risk for developing different kinds of illnesses, including stress-related psychiatric disorders.

In stress conditions, corticotropin-releasing factor (CRF) and arginine vasopressin (AVP) are released from the paraventricular nucleus (PVN) located in the hypothalamus. These peptides travel through the pituitary portal system and act synergistically to stimulate the release of the adrenocorticotropic hormone (ACTH) from the corticotroph cells. Then, ACTH is transported throughout the systemic circulation and binds to receptors in the adrenal cortex of the adrenal gland, resulting in the biosynthesis and release of cortisol [19]. Cortisol can affect multiple organs and biological processes, such as metabolism, growth, inflammation, cardiovascular function, cognition, and behavior [20, 21], by binding to specific receptors in the body and in several brain regions, as the hypothalamus, anterior pituitary and medial prefrontal cortex. The central and peripheral effects of cortisol are mediated by two intracellular glucocorticoid receptor subtypes: the high-affinity type I receptor or mineralcorticoid receptor (MR) and the low-affinity type receptor or glucocorticoid receptor (GR). It has been suggested that MRs have a high affinity for both cortisol and aldosterone; they bind cortisol when it is detectable at low concentrations. The GRs have a relatively low affinity for cortisol, but high affinity for dexamethasone (DEX) [22]; moreover, they bind cortisol at high concentration, reflecting what occurs in stress conditions.

The HPA axis is regulated by an auto-regulatory mechanism mediated by cortisol itself, that is crucial in the maintenance of the homeostatic functions of the HPA axis. Indeed, when cortisol levels rise, as in response to stress, the MRs are saturated and, consequently, cortisol binds the GRs, promoting a cascades of events that represent the main transduction signals of glucocorticoids in stress conditions.

So far, the HPA axis activity has been widely investigated in the context of childhood trauma experiences and findings support alterations in HPA axis in subjects exposed to stress early in life. Indeed, several studies have reported alterations in the cortisol circadian rhythm and levels, indicating a deregulation of the HPA axis responsiveness, due to childhood trauma experiences, upon stress conditions [23–25].

Despite the large amount of data on the HPA axis functionality as consequence of exposure to stress early in life, only a few studies have investigated possible alterations of this axis in BPD patients. For example, higher urinary cortisol levels have been found in BPD patients compared to controls [26, 27].

Southwick and colleagues [26] found higher 24 h urinary cortisol levels in patients with PTSD compared to patients with PTSD and comorbid BPD, suggesting that these alterations might reflect differences in the severity of PTSD symptoms rather than factors related to BPD per se.

Another study [27] explored overnight urinary free cortisol levels showing higher cortisol levels in BPD patients than in controls. A negative association between cortisol and PTSD symptoms was also observed. Moreover, when BPD patients were divided according to the presence of high or low number of PTSD symptoms, very high cortisol levels were found only in BPD patients with a low number of PTSD symptoms. Rinne and collaborators [28] found an exaggerated ACTH and cortisol response during the DEX/CRH test in the blood of BPD female subjects who had experienced childhood abuse. Carvalho Fernando and colleagues [29] investigated the effects of cortisol administration on response inhibition of emotional stimuli in patients with BPD compared to controls. They found that acute cortisol elevations decreased the reaction time to target stimuli in both BPD patients and controls, but they did not differ in task performance.

Also genetic association studies support alterations in HPA axis functionality in association with childhood trauma exposure. Martin-Blanco and collaborators [30] have investigated the contribution of genetic variants within genes in the HPA axis, also in the context of childhood trauma exposures, in a sample of BPD patients and controls. The authors performed a case-control study focusing on 47 SNPs in 10 HPA axis genes. Data showed an association between polymorphic variants within the FK506 Binding Protein 5 (FKBP5) and Corticotropin Releasing Hormone Receptor (CRHR) genes with the diagnosis of BPD. In particular, two FKBP5 polymorphisms, rs4713902 and rs9470079, showed significant association with BPD. Stronger associations were found in patients exposed to childhood trauma where the risk alleles of other two FKBP5 polymorphisms, rs3798347-T and rs10947563-A, were more frequently represented in patients with a history of childhood physical abuse and emotional neglect than in patients who had never experienced these trauma and controls.

All these findings suggest an association between a deregulated functionality of the HPA axis and childhood trauma and highlight the involvement of this biological system in the development of BPD.

#### BPD and neurotransmission

In addition to the presence of HPA axis dysfunction, several studies have also proposed that childhood trauma can affect glutamatergic, serotonergic, dopaminergic and noradrenergic transmission, suggesting that BPD is the result of alterations in several interacting neurotransmitter systems [31, 32].

Glutamatergic and N-methyl-D-aspartate (NMDA) neurotransmissions play a critical role in neurodevelopment, synaptic plasticity, learning and memory [33, 34] and alterations in all these processes have been involved also in the vulnerability and pathophysiology of BPD [35]. For example, neuroimaging studies in BPD patients as compared to controls have consistently demonstrated the presence of decreased synaptic density and volume in several brain regions involved in spatial or autobiographical memory and in the modulation of vigilance and negative emotional states, such as hippocampus and amygdala, which are also enriched in NMDA receptors [36] (see also paragraph “BPD and neuroimaging studies”). Moreover, early chronic stress and mistreatments experienced during life by BPD patients have been found able to impact dendritic arborization, thus contributing to the development of morphological alterations associated with BPD symptoms [37, 38].

The serotonin transporter gene (5-HTTLPR) and its related signaling in neurotransmission represent another system involved in the pathogenesis of BPD [39–42]. In particular, a functional single nucleotide polymorphism (SNP) within this gene (the 5-HTTLPR S/L SNP) has been widely reported to be a modulator of early life stressful events by several studies [43–45]; interestingly, it has been also associated with BPD symptoms [42, 46]. For example, Wagner and collaborators [42] investigated the effects of 5-HTTLPR S/L SNP and of early life stressful events on impulsivity, assessed by the Barratt Impulsiveness Scale (BIS), in BPD patients. The authors reported an association between the presence of stressful events with lower BIS impulsivity scores, suggesting that subjects who have experienced trauma, in particular sexual abuse, may show a reduced impulsivity as a consequence of the activation of coping mechanisms that control behavior and social interaction. Further analyses conducted by the same authors indicated that S-allele carriers showed higher impulsivity scores when exposed to early life stressful events as compared to LL omozygotes, suggesting that patients with 5-HTTLPR S-allele are more vulnerable to early life stress. These data highlight the contribution of the serotonergic system on impulsivity in BPD [42].

Another gene suggested to be a genetic risk factor for BPD is represented by Catechol-O-methyltransferase (COMT), an enzyme catalyzing the degradation of catecholamines, including the neurotransmitters dopamine, epinephrine, and norepinephrine; however, literature data on the role of this gene are contrasting. In a first study conducted by Wagner and collaborators [47], the COMT Val^158^Met SNP has been found associated with early life stressful events and impulsive aggression, assessed by the Buss-Durkee-Hostility Inventory (BDHI) sum score, in female BPD patients. In particular, the authors identified that in COMT Val^158^Val carriers, but not in Val/Met and Met/Met carriers, childhood sexual abuse and the cumulative number of stressful events were associated with lower BDHI impulsive aggression scores. However, in another study conducted by the same authors, the effect of the COMT Val^158^Met SNP on the association between stressful life events and impulsivity was not confirmed [48], probably due to the small sample size. The same authors [49] also investigated, in a group of BPD patients and controls, the role of (i) the COMT Val^158^Met SNP, (ii) the 5-HTTLPR S/L variant and (iii) their interaction as genetic vulnerability factors for BPD. Data showed that the genotype COMT Met^158^Met was over-represented in BPD patients than in controls, whereas no differences in 5-HTTLPR genotype between BPD and controls were reported. In addition, the COMT Met^158^Met genotype was significantly over-represented in BPD patients carrying at least one 5-HTTLPR S-allele and, interestingly, an interaction between the COMT Met^158^ and the 5-HTTLPR S-allele was also observed. These results suggest an interactive effect of COMT and 5-HTTLPR gene variants on the vulnerability to develop BPD and, according to the authors, highlight again the key role of the serotonergic and dopaminergic system in the pathogenesis of BPD.

Martin-Blanco and collaborators [50] investigated the possible involvement of the noradrenergic system in BDP pathogenesis, by evaluating genetic variants within 4 noradrenergic genes. In addition to COMT, the authors selected Dopamine Beta-Hydroxylase (DBH), that acts transforming dopamine into noradrenaline, Solute Carrier Family 6 Member 2 (SLC6A2), a transporter responsible for the reuptake of extracellular neurotransmitters, and Adrenoceptor Beta 2 (ADRB2), that mediates the catecholamine-induced activation of adenylate cyclase through the action of G proteins. The authors’ findings indicated that only genetic variants within 3 genes (COMT, DBH and SLC6A2) were associated with an enhanced risk to develop BPD.

These studies, taken together, show that alterations in several neurotransmitter systems could be involved in BPD pathogenesis; however, due to the small number of available studies, further investigations are needed.

#### BPD and the endogenous opioid system

According to Bandelow and Schmahl’s theory, a reduction in the sensitivity of the opioid receptors or in the availability of endogenous opioids might constitute part of the underlying pathophysiology of BPD [51].

Endogenous opioids mainly include three classes (endorphins, enkephalins and dynorphins), which activate three types of G protein-coupled receptors (μ, δ, and κ opioid receptors [52]). One of the most important endogenous opioid is β-endorphin which is synthesized in part in the arcuate nucleus of the hypothalamus and is released into the blood, the spinal cord and in various brain regions, including reward-related areas [53]. β-endorphin is activated by a variety of stressors [54] and induce euphoria and analgesic effects (for example during childbirth and during positive experiences [55]).

The μ-opioid receptors appear to be more relevant for the social and affective regulation associated with BPD, suggesting that this system can contribute to the interpersonal vulnerabilities and intrapersonal pain of BPD. These receptors are widely distributed throughout the human Central Nervous System (CNS), with a particular density in the basal ganglia, cortical structures, thalamic nuclei, spinal cord, and specific nuclei in the brainstem [56].

The endogenous opioid system modulates responses to acute and chronic stressful and noxious stimuli that induce physical, emotional, or social pain. In animal models, the endogenous opioid system has been implicated in affiliative responses, emotion and stress regulation, including stress-induced analgesia and impulsive-like behavior [57]. Using the mother-infant separation paradigm in rhesus monkeys, Kalin and collaborators [57] studied for the first time the role of the opioid system in modulating the behavioural and neuroendocrine consequences of a brief occurring stressor. The authors conducted several experiments where animals received morphine, an opioid agonist, naloxone, an opioid antagonist or both to test the increase in vocalization and the activation of the HPA axis in infant primates separated or not from their mothers. The results showed that morphine significantly decreased separation-induced vocalizations and locomotion without affecting activity levels, whereas naloxone increased separation-induced vocalizations and environmental exploration. When the two drugs were co-administered, the effect of morphine was reversed only with the 0.1 mg/kg dose of naloxone. The authors also assessed the effects of separation on neuroendocrine function and tested whether activation of the opioid system may attenuate these effects by measuring plasma concentrations of ACTH and cortisol in infant rhesus monkeys separated or not separated from their mothers, treated with morphine or naloxone or co-treated with the two drugs. Plasma ACTH and cortisol levels were higher in infant rhesus monkeys separated from their mothers compared to not separated ones, confirming the involvement of the HPA axis during stress exposure. However, only ACTH plasma levels were modulated by morphine and by naloxone and by their interaction in the group of infant separated by their mothers. These findings suggest that the endogenous opioid system is involved in mediating separation-induced vocalizations and influences the HPA axis activation following a stress condition.

In humans, regional endogenous opioid system activation has been associated with suppression of both sensory and affective qualities of stressors and with trait impulsivity [58–60] whereas its regional deactivation has been related to hyperalgesic responses and increases in negative affect during stress [61]. The hypothesis is that the activation of the μ-opioid receptors could have a suppressive effect during emotional or physical challenges that threaten organism homeostasis.

Research has described regional alterations in the function of the endogenous opioid system and μ-opioid receptors in brain regions involved in emotion and stress processing, decision making, and pain and neuroendocrine regulation. However, to date, there is only limited evidence of alterations of endogenous opioid levels in BPD patients. In an interesting study Prossin and collaborators [61] investigated the role of the endogenous opioid system and μ-opioid receptors in emotion regulation in un-medicated female BPD patients compared to female controls by using positron emission tomography (PET) (see paragraph “BPD and neuroimaging studies” for details).

Comparing BPD patients to their matched controls, the authors found significant differences in baseline regional μ-opioid receptor concentrations in vivo, as well as in this neurotransmitter system’s response to a negative emotional challenge that can be related to some of the clinical characteristics of BPD.

### BPD and neuroimaging studies

#### Volumetric alterations in brain areas involved in stress response

To date, several functional and structural in vivo neuroimaging studies have been performed in BPD patients, detecting alterations mainly localized in the limbic circuit and in frontal cortex. These regions are related to the distinctive clinical features of the disorder (i.e impulsivity, aggression, and emotional reactivity). The most replicated result, confirmed in recent meta-analyses [37, 62, 63], is represented by the reduction in the volumes of the hippocampus and the amygdala of BPD patients compared to controls [36, 64–69]. The robustness of this finding seems to suggest that volumetric decreases in these two brain areas could be specific for BPD and thus useful as possible endophenotypes of illness. In 2000 Driessen and collaborators [36] performed the first magnetic resonance imaging volumetric measurement of the hippocampus, amygdala, temporal lobes, and prosencephalon in 21 female BPD patients and female controls, reporting in BPD patients a volume reduction of the 16% in the hippocampus and of the 8% in the amygdala. Moreover, hippocampal volumes were negatively correlated with the extent and the duration of self-reported early trauma, but only in the entire sample of BPD patients and controls.

The role of PTSD and trauma as comorbidity with BPD on hippocampus and amygdala volumes has been object of investigation but the results are still controversial. Schmahl and colleagues [38] compared two groups of un-medicated BPD female patients with and without comorbid PTSD and 25 female controls. They found reduced hippocampal volumes only in patients with BPD and comorbid PTSD but not in BPD patients without a history of PTSD as compared to controls. Similarly, Kreisel and collaborators [70] investigated in details the hippocampal structural volumes comparing 39 BPD patients with 39 matched controls, and, although no volume differences were found between the two groups, patients with a lifetime history of PTSD had a smaller hippocampal volume (−10,5%) than those without comorbid PTSD. Boen and collaborators [71] investigated the volumes of the Cornu Ammonis (CA) and the Dentate Gyrus (DG), two hippocampal structures prone to morphological changes [72] in response to adverse environmental changes in a group of 18 women with BPD and 21 controls. The authors found that the stress-vulnerable DG-CA4 and CA2–3 subfields were significantly smaller in patients with BPD than in controls. However, they did not identify any significant association between subfield volumes and reported childhood trauma.

In another interesting study, Kuhlmann and collaborators [73] investigated alterations in the grey matter of central stress-regulating structures, including hippocampus, amygdala, anterior cingulate cortex and hypothalamus, in female patients with BPD and controls. The authors also explored whether grey matter volume of these four brain structures was associated with childhood trauma, reporting that patients with BPD showed lower hippocampal volumes than healthy controls, but higher volumes in the hypothalamus. Interestingly, hypothalamic volume correlated positively with a history of trauma in patients with BPD.

Two recent meta-analyses [37, 63] evaluated whether the magnitude of hippocampus and amygdala volume reductions may be associated with state-of-illness factors and psychiatric disorders (i.e. PTSD) which often co-occured with BPD. In the Rodrigues’ meta-analysis, the authors included 7 articles with a total number of 124 patients and 147 controls. They showed that both the left and the right sides of hippocampal volumes were reduced in BPD patients with PTSD when compared to controls. The left hippocampal volume was not significantly smaller in BPD patients without PTSD relative to healthy controls and the right hippocampal volume was reduced in patients with BPD without comorbid PTSD, but to a lesser degree than in BPD patients with PTSD. In contrast, the results reported by Ruocco’s meta-analysis [37] which included 11 studies with a total number of 205 BPD patients and 222 controls, revealed that bilateral volume reductions of the amygdala and hippocampus were unrelated to comorbid Major Depressive Disorder (MDD), PTSD, or substance use disorders.

Taken together, all these studies show that the main brain regions involved in BPD are those associated to stress and highlight the importance of classifying subgroups of patients with BPD, especially taking into account the presence of comorbidity with PTSD or of a history of childhood trauma. Notwithstanding, the association between the volume reduction and the degree to which childhood trauma could be responsible for these changes remains unclear.

#### Endogenous opiod system alterations in brain regions involved in stress response

Despite a large amount of data referred to volumetric and morphological alterations in brain regions associated to specific clinical features of BPD, not many neuroimaging studies have been conducted to investigate the role of the endogenous opioid system in BPD. As previously mentioned, Prossin and collaborators [61] measured the in vivo availability of the μ-opioid receptors (non-displaceable binding potential (BPND)) in a group of un-medicated female BPD patients compared to female controls by using PET and the selective radiotracer [11C] carfentanil at baseline and during sustained sadness states. Patients had greater regional μ-opioid BPND than controls at baseline (neutral state) in the left nucleus accumbens, the hypothalamus, and the right hippocampus/parahippocampus relative to comparison subjects, showing an endogenous opioid system activation. As suggested by the authors, differences between BPD patients and controls in baseline in vivo μ-opioid receptor concentrations and in the endogenous opioid system response to a negative emotional challenge can be related to some of the clinical characteristics of BPD patients. These findings show alterations in the function of the endogenous opioid system and μ-opioid receptors in brain regions involved in emotion and stress processing, decision making, and pain and neuroendocrine regulation, features also associated with BPD.

### BPD and epigenetic mechanisms

The influence of environmental factors, such as childhood trauma, has been suggested to occur through epigenetic mechanisms, which may underlie gene-environment associated vulnerability to develop stress-related disorders [74] including BPD where childhood trauma history occurs in most of the patients (with a range between 30 and 90%) [7, 9].

Among the most investigated epigenetic mechanisms there are: (i) DNA methylation, which occurs at CG dinucleotides (CpG) and can influence the spatial structure of the DNA and the binding or the repression of specific DNA-binding proteins to the DNA [75], (ii) histone modifications, which influence the condensation of the DNA around histone proteins and regulate the accessibility of functional regions to transcriptional factors [76] and (iii) post-transcriptional regulation by non-coding RNAs such as microRNAs (miRNAs) [77].

All these epigenetic processes and, in particular, changes in DNA methylation have been widely investigated in the context of long-term negative effects of early life stressful events. In non-human primates and in rodents, several paradigms of stress early in life, including maternal separation or prenatal stress have been associated with epigenetic alterations via DNA methylation [78, 79]. For example, non-stressed dams during pregnancy showed increased frequency of licking and grooming in the first week of the puppies’ life that were associated with changes in DNA methylation within the promoter of genes, such as glucocorticoid receptor gene (NR3C1), known to be involved in behavior and neurodevelopment.

The hypothesis is that the quality of maternal care, affected by stress or depression in pregnancy and post-partum [80, 81] could impact, through epigenetic mechanisms, on gene expression and behavioral traits that are maintained throughout life [78].

Recently, McGowan and colleagues [79] examined DNA methylation, histone acetylation and gene expression in a 7 million base pair region of chromosome 18 containing the NR3C1 gene in the hippocampus of adult rat offspring, whose mothers differed in the frequency of maternal care. The authors found that the adult offspring of high compared to low maternal care showed a pattern of regions spanning the NR3C1 gene which were differentially methylated and acetylated, highlighting the idea that epigenetic changes, in the context of early life stress, involve alterations in gene-networks rather than in a single or few genes.

Similarly, studies in humans reported similar results as those found in rodents, including the increased methylation levels within the NR3C1 promoter region in subjects who reported a history of early life adverse events [82–84]. For example, in another interesting study, McGowan and collaborators [82] found that in humans the cytosine methylation levels of the NR3C1 promoter were significantly increased in the postmortem hippocampus obtained from suicide victims with a history of childhood abuse as compared with those from suicide victims with no childhood abuse or with control samples. Decreased levels of NR3C1 mRNA were also identified, suggesting an effect of childhood abuse on NR3C1 methylation status and gene expression, independently from suicide.

Several epigenetic studies have been also conducted in control subjects characterized for a history of childhood trauma compared to those with no childhood trauma. In this context, Suderman and colleagues [85] have demonstrated, by using a genome-wide promoter DNA methylation approach, an abuse-associated hypermethylation in 31 miRNAs in a sample of control adult males exposed to childhood abuse. The hypermethylated state for 6 of these miRNAs was consistent with an hypomethylation status of their target genes.

Reduced methylation levels of FKBP5 gene within regions containing functional glucocorticoid responsive elements (GRE) were also found in the blood of control individuals exposed to childhood abuse when compared to subjects without a history of trauma [86]. This demethylation was linked to increased stress-dependent gene transcription followed by a long-term dysregulation of the stress hormone system and a global effect on the function of immune cells and brain areas associated with stress regulation. Thus, according to the authors, the changes in FKBP5 methylation levels might increase the differential responsiveness of FKBP5 to GR activation that can remain stable over time. Moreover, Labontè and colleagues [87] have conducted a genome-wide study of promoter methylation in the hippocampus of individuals with a history of severe childhood abuse and control subjects. Methylation profiles were then compared with corresponding genome-wide gene expression profiles. Among all the differentially methylated promoters, 248 showed hypermethylation whereas 114 demonstrated hypomethylation and genes involved in cellular/neuronal plasticity were among the most significantly differentially methylated.

Despite the contribution of DNA methylation has been extensively investigated in association with childhood trauma in the context of pathologies related to stress, studies on the possible involvement of epigenetic mechanisms in BPD vulnerability are only at their birth. Indeed, only few studies are available. In particular, Martin-Blanco and colleagues, investigated the association between NR3C1 methylation status, history of childhood trauma and clinical severity in blood samples of BPD subjects, showing an association between NR3C1 methylation and childhood trauma, in the form of physical abuse, and a trend towards significance for emotional neglect [88]. Regarding NR3C1 methylation and clinical severity, the authors also found a significant association with self injurious behavior and previous hospitalizations. All these findings support the hypothesis that alterations in NR3C1 methylation can occur early in life as consequence of stress exposure and can persist up to adulthood where subjects with higher NR3C1 methylation levels are also those with enhanced vulnerability to develop BPD.

Above to DNA methylation changes within NR3C1, hypo- or hyper-methylation within other genes have been found to play a key role in mediating the impact of early life stress on the development of stress-related disorders, including BPD [89–92]. For example, in a study conducted by Dammann and colleagues [89] DNA methylation pattern of 14 genes, selected because previously associated with BPD and other psychiatric disorders, (COMT, Dopamine Transporter 1 (DAT1), Gamma-Aminobutyric Acid Type A Receptor Alpha1 Subunit (GABRA1), G Protein Subunit Beta 3 (GNB3), Glutamate Ionotropic Receptor NMDA Type Subunit 2B (GRIN2B), 5-Hydroxytryptamine Receptor 1B (HTR1B), 5-Hydroxytryptamine Receptor 2A (HTR2A), Serotonin Transporter 1 (5-HTT), Monoamine Oxidase A (MAOA), Monoamine Oxidase B (MAOB), Nitric Oxide Synthase 1 (NOS1), NR3C1, Tryptophan Hydroxylase 1 (TPH1) and Tyrosine Hydroxylase (TH)), was analyzed in the whole blood of BPD patients and controls. An increase in the methylation levels of HTR2A, NR3C1, MAOA, MAOB and COMT was observed in BPD patients as compared to controls, suggesting that an increased methylation of CpG sites within these genes may contribute to BPD aetiopathogenesis. Recently, Perroud and colleagues [91] investigated the role of childhood trauma on the methylation status of the Serotonin 3A Receptor (5-HT_3A_R), including several CpGs located within or upstream this gene. They analyzed its association with clinical severity outcomes, also in relation with a functional genetic SNP (rs1062613) within 5-HT_3A_R in adult patients with Bipolar Disorder, BPD, and Attention Deficit Hyperactivity Disorder (ADHD). The results showed that differential 5-HT_3A_R methylation status was dependent on the history of childhood maltreatment and the clinical severity of the psychiatric disorder; this association was not specifically restricted to one specific psychiatric disorders investigated by the authors, but was found in patients who reported the higher severity indexes of childhood maltreatment, mainly represented by BPD patients. In particular, childhood physical abuse was associated with higher 5-HT_3A_R methylation levels, whereas childhood emotional neglect was inversely correlated with CpG1 I methylation levels. As suggested by the authors, these results highlight the need to search for history of childhood maltreatment in patients suffering from psychiatric disorders as these events could be associated with the worse negative outcomes. Moreover, the authors found a modulation of the 5HT_3A_R methylation status by rs1062613 at CpG2 III, where patients carrying the risk CC genotype showed the highest levels of methylation at CpG2 III. Since C allele has been also associated with a lower expression levels of 5HT_3A_R, the authors suggested that increased methylation, due to exposure to childhood maltreatment, could lead to a further decrease in the expression of 5HT_3A_R mRNA.

Aiming to identify novel genes that may exhibit aberrant DNA methylation frequencies in BPD patients, Teschler and collaborators [93] performed a genome-wide methylation analysis in the blood of BPD female patients and female controls. The authors reported increased methylation levels of several genes, including neuronal adaptor proteins (Amyloid Beta Precursor Protein Binding Family A Member 2 (APBA2) and Amyloid Beta Precursor Protein Binding Family A Member 3 (APBA3)), zinc-finger transcription factors (GATA Binding Protein 4 (GATA4)), voltage-gated potassium channel gene (Potassium Voltage-Gated Channel Subfamily Q Member 1 (KCNQ1)), guanine nucleotide exchange factors (Proto-Oncogene MCF-2 (MCF2)), adhesion molecules (Ninjurin 2 (NINJ2)) and G protein-coupled receptors (Trace Amine Associated Receptor 5 (TAAR5)) in BPD samples compared to controls. Similarly, using a whole-genome methylation approach, Prados and colleagues [94] analyzed the global DNA methylation status in the peripheral blood leukocytes of BPD patients with a history of childhood adversity and also in patients with MDD characterized by a low rate of childhood maltreatment. Contrary to Teschler [93], who used control subjects as reference group, in this study the authors used MDD subjects, most of them suicide attempters, thus controlling not only for MDD but also for a history of suicide. The authors also assessed possible correlations between methylation signatures and the severity of childhood maltreatment. Data showed that several CpGs within or near genes involved in inflammatory processes (Interleukin 17 Receptor A (IL17RA)), regulation of gene expression (miR124–3) and neuronal excitability and development/maintenance of the nervous system (Potassium Voltage-Gated Channel Subfamily Q Member 2 (KCNQ2)) were differentially methylated, either in BPD compared with MDD or in relation to the severity of childhood maltreatment.

In a more recent study, Teschler and collaborators [95] have analyzed also DNA methylation patterns of the ribosomal RNA gene (rDNA promoter region and 5′-external transcribed spacer/5′ETS) and the promoter of the proline rich membrane anchor 1 gene (PRIMA1) in peripheral blood samples of female BPD patients and controls. The authors have identified a significant aberrant methylation of rDNA and PRIMA1 in the group of BPD patients. Specifically, the average methylation of 6 CpG sites in the promoter of PRIMA1 was 1.6-fold higher in BPD patients compared to controls. In contrast, the methylation levels of the rDNA promoter region and the 5′ETS were significantly lower (0.9-fold) in patients with BPD compared to controls. Furthermore, decreased methylation levels were found for nine CpGs located in the rDNA promoter region and for 4 CpGs at the 5′ETS in peripheral blood of patients compared to controls. These results suggest that aberrant methylation of rDNA and PRIMA1 could be associated with the pathogenesis of BPD.

Taken together, all these studies reveal a complex interplay between BPD, early-life stressful adversities and epigenetic signatures.

### BPD and neuroplasticity (the role of BDNF)

Neuroplasticity refers to brain-related mechanisms associated with the ability of the brain to perceive, adapt and respond to a variety of internal and external stimuli [96, 97], including stress.

The exposure to acute stressful challenges can induce several beneficial and protective effects for the body, which responds to almost any sudden, unexpected events by releasing chemical mediators – i.e. catecholamines that increase heart rate and blood pressure – and help the individual to cope with the situation [20, 98–101]. However, a chronic exposure to stress and thus a chronic exposure to glucocorticoids can have negative and persistent effects on the body, including altered metabolism, altered immunity, enhanced inflammation, cognitive deficits, and also an enhanced vulnerability for psychiatric disorders and for medical conditions such as cardiovascular disease, metabolic disorders and cancer [102, 103].

Neurotrophic factors, and in particular the neurotrophin Brain-Derived Neurotrophic Factor (BDNF), have been identified as key mediators of stress on neuronal connectivity, dendritic arborization, synaptic plasticity and neurogenesis [104–107]. Since its crucial role in brain development and brain plasticity, BDNF has been widely investigated also in several psychiatric diseases, including BPD [108].

For example, Koenigsberg and colleagues [109] found a decrease of Protein Kinase C (PKC) isoenzyme, which is a molecule downstream the activation of BDNF, and BDNF protein levels in the blood of BPD patients, suggesting an alteration of BDNF signaling and consequently of neuroplasticity-related mechanisms in BPD. In another study, Tadic and collaborators [49] investigated the association between BPD and genetic variants within HTR1B and BDNF genes. Although data showed no significant differences in genotype or haplotype distribution for both HTR1B and BDNF variants between BPD patients and controls, logistic regression analyses revealed an association between the HTR1B A-161 variant and the functional BDNF 196A allele in BPD.

Importantly, several findings have also documented epigenetic modifications on BDNF gene in patients with BPD**,** suggesting that childhood maltreatment in BPD patients can cause long term epigenetic alterations of genes crucially involved in brain functions and neurodevelopment, including BDNF, and that these alterations may contribute to enhanced vulnerability to develop BPD pathology. In this regard, Perroud and collaborators [90] measured the percentage of methylation at BDNF CpG exons I and IV and also plasma BDNF protein levels in subjects with BPD and controls. The authors reported significantly higher methylation status in both CpG regions in patients than in controls, with the number of childhood trauma exposures associated with the high levels of BDNF methylation. Moreover, BPD patients had significantly higher BDNF plasma protein levels than controls, but this increase was not associated with changes in BDNF methylation status. More recently, Thaler and collaborators [92] analyzed DNA methylation patterns in the promoter region of BDNF gene in women with bulimia nervosa and with history of BPD and/or trauma events. They reported that bulimia nervosa was associated per se with an hypermethylation within BDNF promoter region sites. This was particularly evident when co-occurring with childhood abuse or BPD.

Overall, these studies support the hypothesis that childhood trauma could be associated with changes in BDNF epigenetic signature, that in turn could contribute to alter cognitive functions in BPD patients. Indeed, higher levels of gene methylation are commonly accompanied by a reduced gene expression. Thus higher BDNF methylation levels should determine reduced expression of BDNF gene and reduced BDNF mRNA levels are widely observed in patients with psychiatric diseases [110–112].

## Conclusions

Up to now, neither a specific gene variant or biological mechanism has been exclusively associated with BPD, but the onset of this disorder has been suggested to depend on the combination of a vulnerable genetic background with adverse environmental factors during childhood.

Among the biological systems found involved in BPD pathogenesis and particularly affected by childhood trauma events, there are: the HPA axis, the neurotransmission mechanisms, the endogenous opioid system and the neuroplasticity. In line with the involvement of these processes, neuroimaging studies in BPD patients have shown volume reductions in the hippocampus and amygdala, both brain regions mainly involved in stress responses, cognition, memory and emotion regulation and an increase in the μ-opioid receptors in the same areas.

Among the environmental factors, early life stressful events, in particular childhood trauma, have been proposed to negatively impact brain development through epigenetic mechanisms. Although a complex interplay between BPD, early-life stressful adversities and epigenetic signatures has been suggested, further investigations are needed in order to better understand the role of genetic background and traumatic events during childhood in the onset of BPD. A better comprehension of these interactions could allow to identify at risk subjects, who could be treated with preventive therapies, such as psychotherapy, and to prevent or minimize the development of the disease later in life.

## Abbreviations

5-HT_3A_R: Serotonin 3A Receptor
5-HTT: Serotonin Transporter 1
5-HTTLPR: Serotonin transporter gene
ACTH: Adrenocorticotropic Hormone
ADHD: Attention Deficit Hyperactivity Disorder
ADRB2: Adrenoceptor Beta 2
APBA2: Amyloid Beta Precursor Protein Binding Family A Member 2
APBA3: Amyloid Beta Precursor Protein Binding Family A Member 3
AVP: Arginine Vasopressin
BDHI: Buss-Durkee-Hostility Inventory
BDNF: Brain-Derived Neurotrophic Factor
BIS: Barratt Impulsiveness Scale
BPD: Borderline Personality Disorder
CA: Cornu Ammonis
CNS: Central Nervous System
COMT: Catechol-O-methyltransferase
CpG: CG dinucleotides
cPTSD: complex Post-Traumatic Stress Disorder
CRF: Corticotropin-Releasing Factor
CRHR: Corticotropin Releasing Hormone Receptor
DAT1: Dopamine Transporter 1
DBH: Dopamine Beta-Hydroxylase
DESNOS: Disorders of Extreme Stress Not Otherwise Specified
DEX: Dexamethasone
DG: Dentate Gyrus
FKBP5: FK506 Binding Protein 5
GABRA1: Gamma-Aminobutyric Acid Type A Receptor Alpha1 Subunit
GATA4: GATA Binding Protein 4
GNB3: G Protein Subunit Beta 3
GR: Glucocorticoid Receptor
GRE: Glucocorticoid Responsive Elements
GRIN2B: Glutamate Ionotropic Receptor NMDA Type Subunit 2B
HPA axis: Hypothalamic-Pituitary-Adrenal axis
HTR1B: 5-Hydroxytryptamine Receptor 1B
HTR2A: 5-Hydroxytryptamine Receptor 2A
IL17RA: Interleukin 17 Receptor A
KCNQ1: Potassium Voltage-Gated Channel Subfamily Q Member 1
KCNQ2: Potassium Voltage-Gated Channel Subfamily Q Member 2
MAOA: Monoamine Oxidase A
MAOB: Monoamine Oxidase B
MCF2: Proto-Oncogene MCF-2
MDD: Major Depressive Disorder
miRNAs: microRNAs
MR: Mineralcorticoid Receptor
NINJ2: Ninjurin 2
NMDA: N-methyl-D-aspartate
NOS1: Nitric Oxide Synthase 1
NR3C1: Glucocorticoid receptor gene
PET: Positron Emission Tomography
PKC: Protein Kinase C
PRIMA1: Prolin Rich Membrane Anchor 1
PTSD: Post-Traumatic Stress Disorder
PVN: Paraventricular Nucleus
SLC6A2: Solute Carrier Family 6 Member 2
SNP: Single nucleotide polymorphism
TAAR5: Trace Amine Associated Receptor 5
TH: Tyrosine Hydroxylase
TPH1: Tryptophan Hydroxylase 1
